# Effects of Atmospheric Aging Processes on Nascent Sea Spray Aerosol Physicochemical Properties

**DOI:** 10.1021/acsearthspacechem.2c00258

**Published:** 2022-10-25

**Authors:** Chathuri P. Kaluarachchi, Victor W. Or, Yiling Lan, Elias S. Hasenecz, Deborah Kim, Chamika K. Madawala, Glorianne P. Dorcé, Kathryn J. Mayer, Jonathan S. Sauer, Christopher Lee, Christopher D. Cappa, Timothy H. Bertram, Elizabeth A. Stone, Kimberly A. Prather, Vicki H. Grassian, Alexei V. Tivanski

**Affiliations:** † Department of Chemistry, University of Iowa, Iowa City, Iowa 52242, United States; ‡ Department of Chemistry and Biochemistry, University of California, San Diego, La Jolla, California 92093, United States; § Scripps Institution of Oceanography, University of California, San Diego, La Jolla, California 92093, United States; ∥ Department of Civil and Environmental Engineering, University of California, Davis, California 95616, United States; ⊥ Department of Chemistry, University of Wisconsin−Madison, Madison, Wisconsin 53706, United States

**Keywords:** atomic force microscopy, aged sea spray aerosol, morphology, phase state, water uptake, composition, particle-to-particle variability

## Abstract

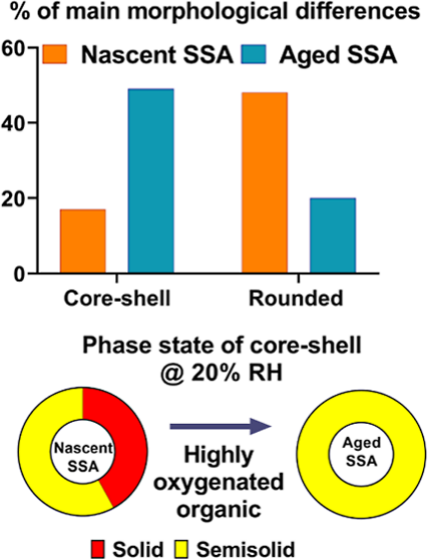

The effects of atmospheric aging on single-particle nascent sea spray aerosol (nSSA) physicochemical properties, such as morphology, composition, phase state, and water uptake, are important to understanding their impacts on the Earth’s climate. The present study investigates these properties by focusing on the aged SSA (size range of 0.1–0.6 μm) and comparing with a similar size range nSSA, both generated at a peak of a phytoplankton bloom during a mesocosm study. The aged SSAs were generated by exposing nSSA to OH radicals with exposures equivalent to 4–5 days of atmospheric aging. Complementary filter-based thermal optical analysis, atomic force microscopy (AFM), and AFM photothermal infrared spectroscopy were utilized. Both nSSA and aged SSA showed an increase in the organic mass fraction with decreasing particle sizes. In addition, aging results in a further increase of the organic mass fraction, which can be attributed to new particle formation and oxidation of volatile organic compounds followed by condensation on pre-existing particles. The results are consistent with single-particle measurements that showed a relative increase in the abundance of aged SSA core–shells with significantly higher organic coating thickness, relative to nSSA. Increased hygroscopicity was observed for aged SSA core–shells, which had more oxygenated organic species. Rounded nSSA and aged SSA had similar hygroscopicity and no apparent changes in the composition. The observed changes in aged SSA physicochemical properties showed a significant size-dependence and particle-to-particle variability. Overall, results showed that the atmospheric aging can significantly influence the nSSA physicochemical properties, thus altering the SSA effects on the climate.

## Introduction

Nascent sea spray aerosols (nSSA) are generated upon the bursting of air bubbles at the ocean–air interface and represent a significant fraction of natural aerosol mass concentration in the atmosphere.^1−4^ During air bubble bursting, organic, inorganic, and biological species from the seawater and sea surface microlayer (SML) can be transferred into the nSSA.^1,3−15^ Therefore, the chemical complexity of nSSA can significantly vary due to the composition and biological activity in the seawater and SML.^3,4,14−23^ Additionally, the differences in formation mechanisms (i.e., film drops vs jet drops) of nSSA at the ocean–air interface can further control their size-dependent and particle-to-particle variability in chemical complexity.^5,14,16,24^ nSSA can influence the Earth’s radiative budget directly, via scattering and absorbing incoming solar radiation, and indirectly by acting as cloud condensation nuclei (CCN) or ice nuclei (IN).^1,6,7,13,25−35^ The surfaces of aerosols can facilitate atmospheric aging with gaseous phase oxidants (i.e., OH radicals and ozone).^36−44^ For example, studies conducted on atmospherically relevant organic model systems showed that atmospheric aging can lead to the formation of new particles,^2,45^ oxidation of volatile and semi-volatile organic compounds from pre-existing particles,^36−38,46−48^ and condensation of oxidized organics on pre-existing particles to form coatings.^36,46−49^ Thus, atmospheric aging can alter the nSSA’s physicochemical properties (i.e., morphology, composition, phase states, and water uptake) and influence on their direct and indirect aerosol effects on the climate.^36−38,46,50^

The effects of aging on the physicochemical properties of aerosols have been studied previously using laboratory-generated model systems. For example, atmospheric organic aerosol proxies (e.g., alkanes and oxidized organic compounds) showed a formation of highly oxygenated organic compounds with increasing atmospheric aging time (i.e., increasing OH exposure).^36,47,48,51^ The morphology of aerosols can have a significant impact on the rates of atmospheric aging.^36^ In particular, core–shells can undergo the OH-initiated aging process within days, compared to rounded particles where the timescale can be weeks or even months.^36,52^ In addition, an increase in hygroscopicity and water uptake efficiency of aerosols have been observed due to the atmospheric aging, which in turn impacts their phase state.^36,43,47,53,54^ The changes in the phase state can change aerosol’s water content, solute concentration, and viscosity that subsequently alter their direct and indirect effects.^1,2,11,42,55−58^ However, to our knowledge, no previous studies had investigated the effects of atmospheric aging on physicochemical properties of single-particle nSSA and their size-dependent particle-to-particle variabilities.

Herein, we investigated the effects of atmospheric aging on various physicochemical properties of nSSA (size range of 0.1–0.6 μm) generated during a phytoplankton bloom from the Sea Spray Chemistry And Particle Evolution (SeaSCAPE) mesocosm study in 2019.^1,2^ Aged SSAs were generated by exposing freshly generated nSSA to OH radicals, simulating approximately 4–5 days of atmospheric aging. In addition, the formation of new particles (i.e., secondary marine aerosols) and condensation of oxidized organic compounds on existing particles were observed. Filter-based thermal optical analyses and ion chromatography were used to investigate the size-dependent bulk-ensemble organic mass fraction in nSSA and aged SSA, which provided ensemble-averaged composition data for the aerosol population, with significant organic enrichment observed for aged SSA. To better understand the particle-to-particle variability in nSSA versus aged SSA organic enrichment and their corresponding morphology, phase state, and water uptake properties, single-particle atomic force microscopy (AFM) and AFM–photothermal infrared spectroscopy (AFM–PTIR) analyses were employed. A significant change of these properties was observed due to the atmospheric aging, and results provide an important insight on how the aging influences the physicochemical properties of nSSA.

## Materials and Methods

### Nascent and Aged SSA Generation and Collection for Offline Single-Particle Studies

Nascent SSA (nSSA) were generated throughout a phytoplankton bloom from a wave-simulation channel facility which contained filtered seawater from the southern coast of California, during the Sea Spray Chemistry And Particle Evolution (SeaSCAPE) 2019 study.^1,2^ A micro-orifice uniform deposit impactor (MOUDI; MSP, Inc., model 110) at a flow rate of 30 L/min was used to deposit individual submicrometer nSSA onto hydrophobically coated (Rain-X) silicon substrates (Ted Pella, Inc.) at ∼80% relative humidity (RH).^1^ The MOUDI stages 7, 8, and 9 were used that correspond to 50% cutoff aerodynamic diameter range of 0.32–0.60, 0.18–0.32, and 0.10–0.18 μm, respectively. Additional details of the nSSA generation and deposition can be found elsewhere.^1^ For the purpose of comparison, both nSSA and aged SSA were collected on the same sampling day (peak of the bloom, August 2nd) over the same size range.

A potential aerosol mass oxidation flow reactor (PAM-OFR, Aerodyne Inc) was used to produce hydroxyl (OH) radicals, which can simulate aging of aerosols with atmospheric time-equivalent aging from a fraction of a day to several weeks.^2,59,60^ Here, by using the PAM-OFR, aged SSA were generated by exposing nSSA to OH radicals (average concentration of ∼5.9 × 10^11^ molecules/cm^3^, aerosol residence time of ∼2 min), which corresponds to 4–5 days of atmospheric aging.^2,59^ Before aging, nSSA stream was passed through a denuder (CARULITE-200, Ozone Solutions) to remove ozone (O_3_) from the wave-channel headspace.^2^ Additional details of the aged SSA generation using the PAM-OFR can be found elsewhere.^2^ The PAM-OFR sampled nSSA from a headspace of the wave-channel to generate aged SSA.^1,2^ However, in addition to aged SSA, new particle formation (i.e., secondary marine aerosols, SMA, typical particle diameter < 100 nm) was also observed, likely as a result of oxidation and condensation of volatile organic compounds from the wave-channel headspace.^2^ In the present work, atomic force microscopy (AFM) single-particle analysis was limited to particle sizes above 100 nm, thus largely excluding SMA particles that were <100 nm in size. In addition, we note that the composition of aged SSA studied here may be somewhat influenced by condensation of oxidized volatile or semi-volatile organic compounds onto pre-existing particles in PAM-OFR. The generated aged SSA were deposited onto hydrophobically coated silicon substrates using MOUDI (MSP, Inc., model 125R, flow rate 10 L/m) stages of 7, 8, and 9 at ∼20% RH. All samples were stored in clean Petri dishes and kept inside a laminar flow hood (NuAire, Inc., NU-425-400) at ambient temperature (20–25 °C), 20–25% relative humidity range, and pressure for 2–4 months prior to AFM and AFM–PTIR experiments.

### AFM Imaging to Determine the Morphologies and Organic Volume Fraction of Aged SSA Core–Shell at 20% RH

Particle locations for single-particle imaging and analysis were selected in a completely random and unbiased manner.^1^ A molecular force probe 3D AFM (Asylum Research, Santa Barbara, CA) was used for imaging individual substrate-deposited aged SSA at ambient temperature (20–25 °C) as described in prior studies.^1,11,55,61^ A custom-made humidity cell was used to control RH with a range of 20–80%.^11^ Prior to the AFM measurements at a particular RH, at least 10 min of equilibrium time was allocated to ensure that aged SSA are in thermodynamic equilibrium with surrounding water vapor.^11,55,61^ Silicon nitride AFM tips (MikroMasch, model CSC37, typical tip radius of curvature of ∼10 nm, nominal spring constant of 0.5–0.9 N/m) were used for imaging and force spectroscopy measurements.^11,55,61^ AFM AC (intermittent contact) mode imaging was used to collect 3D height and phase images of individual aged SSAs to determine their morphology and volume-equivalent diameter and quantify the organic volume fraction (OVF) and corresponding organic coating thickness (OCT) for core–shell aged SSA, as described in prior studies.^11,55,61^ The OVF is defined as the ratio of the shell volume (assumed predominantly organic) to the total particle volume, while the OCT represents the projected thickness of organic coating around the inorganic core.^1,11,61,62^ By assuming the core is predominantly inorganic and the shell primarily organic, the single-particle OVF represents the amount of organic present in the particle relative to the total particle volume.^1,11,61^ For the morphological analysis, approximately 100 individual aged SSAs were investigated, while for the OVF and OCT analyses, 10 or more individual aged SSA core–shell within each size range were investigated. The relative abundance of identified morphological categories (rounded, core–shell, prism-like, rod–shell, and aggregate) and OVF and OCT values were recorded as an average and one standard deviation at three volume-equivalent diameter ranges of 0.10–0.18, 0.18–0.32, and 0.32–0.60 μm. The observed aged SSA morphology, OVF, and OCT were compared with nSSA sample collected on same sampling day over the same three size ranges.^1^

Because the total number of individual particles that can be practically studied with AFM is limited, we utilized a statistical probability distribution analysis to assess the statistical significance of the AFM-based morphology and phase state measurements.^1^ The detailed description about the approach can be found elsewhere.^1,63,64^ Briefly, the probability distributions associated with the likelihood of sampling one of the five morphologies, or one of the three phase states, were generated using a self-coded Monte Carlo-like simulation method for a “true” population of 10,000 particles.^1,63−65^ The average with one standard deviation for the fraction of particles from each morphological type or phase states were obtained by fitting the probability distribution plots with the Gaussian function.^1^ The results were recorded for nSSA and aged SSA samples as a function of RH and volume-equivalent diameter range.

### AFM Measurements of Aged SSA Water Uptake and Phase State at RH Range of 20–80%

The analysis of 3D growth factor (GF) at 80% RH was employed to quantify the water uptake properties of aged SSA on a single-particle basis.^1,66−68^ The GF is defined as the ratio of the volume-equivalent diameter of an individual SSA at 80% RH over the corresponding volume-equivalent diameter recorded at 20% RH, where higher values would indicate the presence of more hygroscopic components.^1,66−68^ The GF measurements were performed on approximately 10 individual core–shell and rounded aged SSA at their highest relative occurrence size range of 0.32–0.60 and 0.10–0.18 μm, respectively, and the values were reported as an average and one standard deviation.

The AFM force spectroscopy was employed to identify the phase state at 20 and 60% RH under ambient temperature (20–25 °C) and pressure for aged SSA with the most abundant morphologies (i.e., core–shell, rounded) using a previously reported method.^1,11,55,57^ The RH values were selected as a benchmark based on sucrose that shows solid to semisolid and semisolid to liquid phase transitions at ∼20 and 60% RH, respectively.^1,11,55,57^ A maximum force of 20 nN and scan rate of 1 Hz were used.^1,11^ At least five force plots were collected by probing at the shell region of core–shell and at approximately the center of the rounded aged SSA.^1^ The collected force plots were then used to quantify the viscoelastic response distance (VRD, nm) and the relative indentation depth (RID, the ratio of the indentation distance over the particle height) for an individual particle.^11,55^ The single-particle phase state identification was conducted using an established framework from VRD and RID measurements, as described in prior studies.^1,11,55,57^ The VRD values measured on aged SSA in the semisolid phase state were reported as an average and one standard deviation. Approximately 10 or more individual aged SSAs for each morphology were investigated. The VRD values and relative abundance (i.e., an average and one standard deviation for fraction of particles) of phase states for the shell of core–shell SSA and rounded particles were recorded at three volume-equivalent diameter ranges of 0.10–0.18, 0.18–0.32, and 0.32–0.60 μm. The observations on phase states and water uptake of aged SSA were compared with the nSSA results reported previously.^1^

### AFM–PTIR Measurements of Aged SSA Composition at ∼20–30% RH

AFM–PTIR spectroscopic measurements were collected using a commercial AFM-IR microscope (nanoIR2, Bruker) with a tunable mid-IR quantum cascade laser (QCL MIRcat-QT, Daylight solutions). Images and spectra were collected at ∼20–30% RH and ambient temperature (23–26 °C) and pressure on individual aged SSA deposited on silicon substrates placed on MOUDI stages 7, 8, and 9. Analysis was conducted using silicon nitride probes with a chromium-gold coating (HQ: NSC19/CR-AU, MikroMasch, typical tip radius of curvature 35 nm, and a nominal spring constant range of 0.05–2.3 N/m). AFM imaging was conducted in the tapping mode at a scan rate of 0.5 Hz. AFM–PTIR spectra were collected with a nominal spatial resolution below 35 nm and a spectral resolution of 5 cm^–1^, co-averaging over 128 laser pulses per wavenumber.^1^ A reference spectrum was taken on the substrate and subtracted from all corresponding spectra obtained on individual particles. For core–shell-aged SSA, spectra were taken at the core and shell particle regions, while for rounded aged SSA spectra were taken at an approximate center of each particle. Even accounting for differences in morphology, the large diversity of spectra between the aged SSA is reflected in large variances between particles. The PTIR results collected on aged SSA were compared with the nSSA results, which were recorded in a prior study.^1^

### Bulk Measurements of nSSA and Aged SSA Size-Dependent Organic and Inorganic Mass Fractions

For these measurements, nSSA and aged SSA samples were collected simultaneously during the peak of the phytoplankton bloom using five stage SIOUTAS Personal Cascade Impactors (PCIS, SKC model 225–370; 50% aerodynamic diameter range cutoff for each stage).^1^ The top four stages consisted of pre-baked 25 mm Al foil disks (0.25–0.50, 0.50–1.0, 1.0–2.5, and >2.5 μm) and the last stage a pre-baked 37 mm quartz fiber filter (QFF, PALL Life Sciences, <0.25 μm). The nSSA were collected directly from the wave flume and aged SSA by first oxidizing in the PAM-OFR using the conditions described above prior to collection.^1,2^ Flow rates of 9 L/min and ∼75–95% RH were maintained, and all samples were stored frozen at −20 °C until the analysis. Organic carbon (OC) was measured via a thermal optical analyzer (Sunset Laboratories, Forest Grove, OR) and common inorganic ions were separated and quantified via high-performance ion exchange chromatography with conductivity detection (Dionex ICS5000, Sunnyvale, CA).^12,69,70^ A stainless-steel punch was used to sub sample substrates, which were subsequently extracted in 4 mL of ultrapure water (>18.2 MΩ·cm, Thermo Barnstead Easy Pure II) and filtered (0.45 μm polypropylene, Whatman). Inorganic mass was estimated as the sea salt using the measured mass of sodium converted to the mass of the sea salt via a sodium/sea salt ratio of 3.26, as described previously.^71^

## Results and Discussion

### Impact of Atmospheric Aging on Bulk Organic Enrichment in nSSA

**Figure 1:**
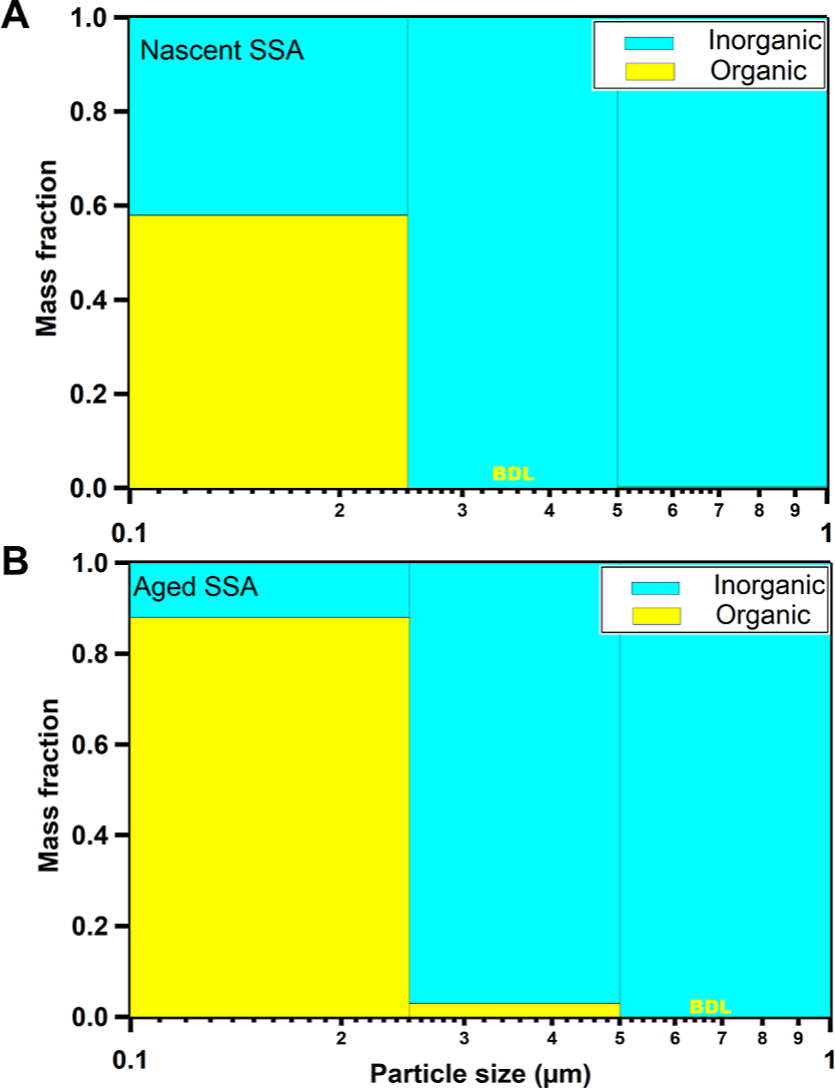
Organic (yellow) and inorganic (cyan) mass fractions versus particle size for (A) nascent and (B) aged SSA samples. The width of each bar indicates the SIOUTAS Personal Cascade Impactor cutoff size range at 74–96% RH. BDL indicates the measured organic mass fraction was below the detection limit. Organic and inorganic mass fractions for nascent SSA were adapted from Kaluarachchi et al., 2022.^1^ Copyright 2022 American Chemical Society.

The bulk ensemble-averaged method that was previously reported was used to investigate the size-dependent organic enrichment in SSA samples.^1,61^Figure 1A,B shows the size-dependent bulk organic and inorganic mass fractions in nSSA and aged SSA, respectively. Both samples showed an increase in the organic mass fraction with decreasing particle size, consistent with previous mesocosms and laboratory studies on nSSA.^1,2,61^ The relative uncertainty in the organic mass fraction measurement for the smallest size range nSSA and aged SSA were estimated to be 30–34 and ∼30%, respectively. In addition, atmospheric aging increases the organic mass fraction in aged SSA across all sizes. Aged SSA with sizes below 0.25 μm showed the highest (∼30%) increase of organic mass fraction relative to nSSA of similar sizes. The overall relative increase in the organic mass fraction can be attributed to the oxidation of volatile organic compounds in PAM-OFR followed by condensation on pre-existing particle or new particle formation.^1,2,36,38,39,72^ An increase in the organic mass fraction and possible changes in the composition of organic compounds in aged SSA as a result of atmospheric aging are expected to influence their physical–chemical properties, such as water uptake and phase state.^3,39,43,44,50,72^ For example, studies have shown that aging can result in an increase of oxygenated functional groups (e.g., hydroxyls and carbonyls) on parent particles, which in turn increases their hygroscopicity.^39,72,73^ Noteworthy, the size-dependent bulk organic enrichment in aged SSA relative to nSSA provides an ensemble-averaged value of an entire population of aerosols within a particular size range; however, it does not fully explain the origin of such enrichment nor provide an assessment on a possible particle-to-particle variability in the organic enrichment. Thus, single-particle measurements were next utilized to further assess the effects of aging on the nSSA composition and morphology and then supplemented with single-particle phase state and water uptake measurements.

### Impact of Atmospheric Aging on Size-Dependent Morphological Distribution of nSSA

**Figure 2:**
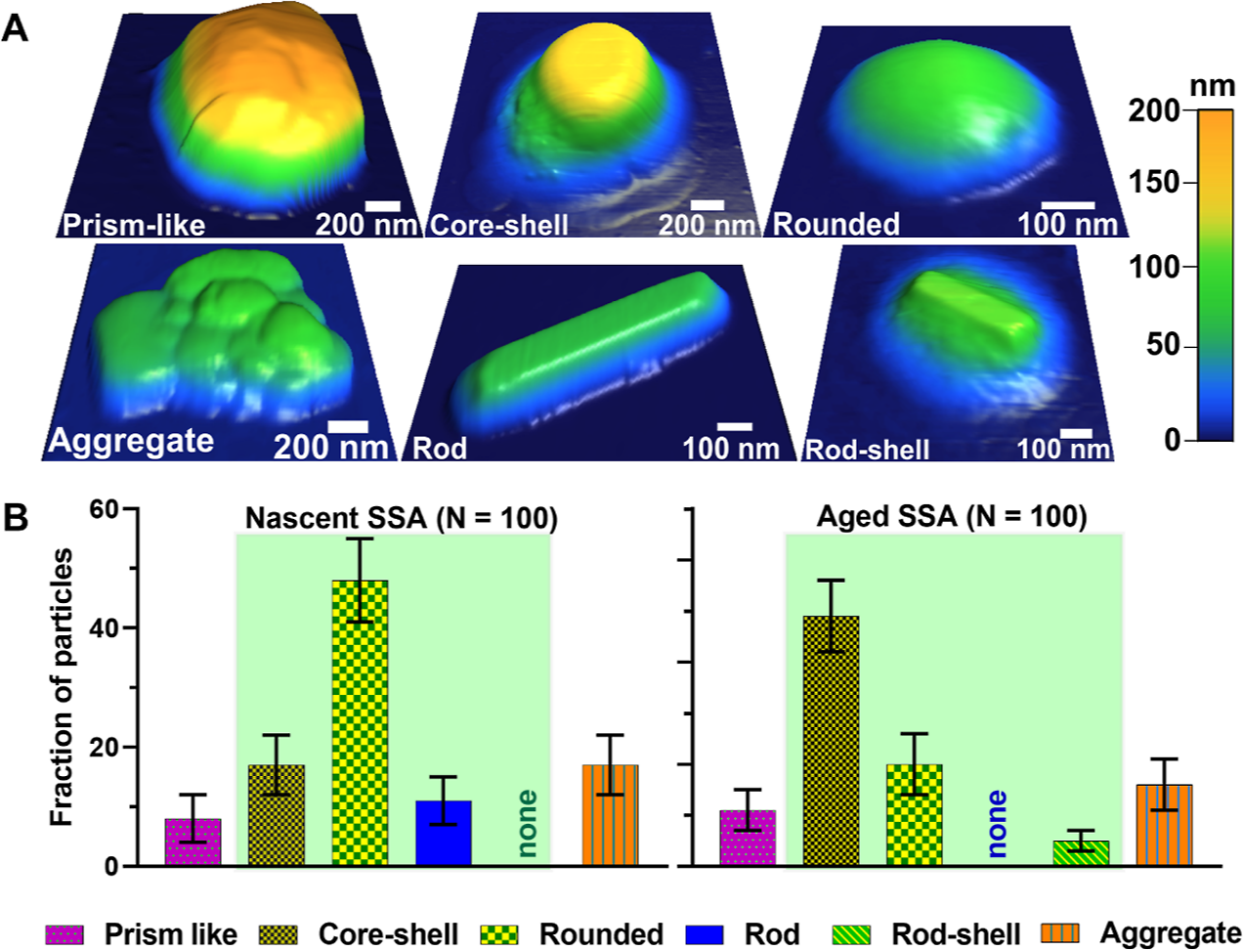
(A) Selected illustrative AFM 3D height images of six observed morphological categories (prism-like, core–shell, rounded, rod, rod–shell, and aggregate) for nascent and aged SSA samples. The maximum height range is 200 nm for each image. (B) Average and one standard deviation of fraction of particles (%) from six morphological categories for nascent (left) and aged (right) SSA for a total number of particles (*N*) 100 within the volume-equivalent diameter range of 0.10–0.60 μm. The term “none” indicates the absence of a particular morphology type (i.e., no rod–shells for nascent SSA, and no rods for aged SSA). Statistically significant differences of morphological categories are highlighted by green areas. AFM 3D-height image of rod and rounded SSA, and histogram for nascent SSA was adapted from Kaluarachchi et al., 2022.^1^ Copyright 2022 American Chemical Society.

AFM single-particle imaging at ∼20% RH was used to investigate substrate-deposited aged SSA morphologies. Figure 2A shows representative AFM 3D height images of five main aged SSA morphological categories identified here (prism-like, core–shell, rounded, rod–shell, and aggregate) within a volume-equivalent diameter range of 0.10–0.60 μm. The categorization of morphologies was conducted qualitatively using AFM 3D height and phase images, as described previously.^1,12,55,57,61^ The aged SSA morphologies were compared with nSSA morphologies (prism-like, core–shell, rounded, rod, and aggregate) reported previously for the same sampling day.^1^ Overall, both nSSA and aged SSA samples had particles with core–shell, prism-like, rounded, and aggregate morphologies. However, the rod morphology was only observed for nSSA, while the rod–shell morphology was only observed for aged SSA. As will be shown in the next section using AFM–PTIR, the rod particles were predominantly inorganic sulfates, while the rod–shell had inorganic sulfate rods with a predominantly organic shell. Therefore, it is likely that the organic shell in the rod–shell was formed from the condensation of volatile organic compounds onto pre-existing rods during the aging process of nSSA.

Figure 2B shows the relative distribution of main morphological categories for nSSA versus aged SSA over the same volume-equivalent size range of 0.10–0.60 μm. The relative distribution of each morphological type was assessed by performing statistical probability distribution analysis, as shown in prior studies.^1^ From this analysis, we established statistically significant differences in the relative abundance of four main morphological categories—rounded, core–shell, rod, and rod–shell. Specifically, due to atmospheric aging, the relative abundance of rounded and rod SSA decreased from 48 to 20% and from 11% to none, respectively. In contrast, the relative abundance of core–shell and rod–shell SSA increased from 17 to 49% and from none to 5%, respectively. While the exact origin for the observed morphological changes remains unknown, it is likely originating from a combination of several factors. First, organic compounds in nSSA likely became more oxygenated and some more volatile, which could explain an observed decrease in the relative abundance of rounded SSA that are predominantly organic.^1,39,47,72,74^ Second, semi-volatile or low-volatility organic compounds can condense onto pre-existing particles, leading to the formation of more core–shell particles (i.e., condensation onto prisms or core–shells) or formation of rod–shells (i.e., condensation onto rods).^39,47,74−77^ Third, the formation of more oxygenated organic compounds in aged SSA likely decreases their viscosity, which in turn can facilitate a more defined phase separation of organic and inorganic compounds within substrate-deposited particles, that can be more readily observed with AFM.^46,61,72^ Overall, our single-particle results clearly demonstrate that the atmospheric aging leads to changes in the relative abundance of SSA morphologies with a significant increase in the core–shells.

**Figure 3:**
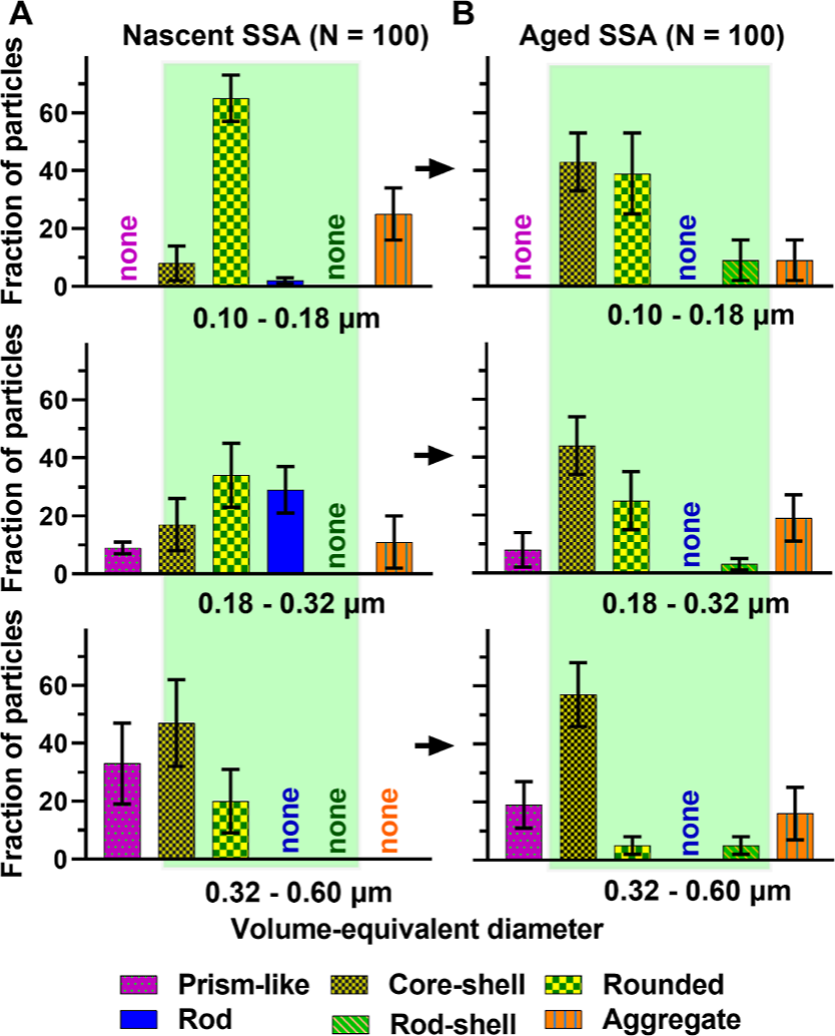
Relative distribution of the observed morphological categories (prism-like, core–shell, rounded, rod, rod–shell, and aggregate) of (A) nascent vs (B) aged SSA for a total number of particles (*N*) of 100, at three selected volume-equivalent diameter ranges of 0.10–0.18, 0.18–0.32, and 0.32–0.60 μm. The term “none” indicates absence of a particular morphology type within a specific subpopulation of SSA. Arrows are for illustrative purposes only and show changes in the morphological distribution from nascent to aged SSA within a particular volume-equivalent diameter range. Statistically-significant differences between nascent and aged SSA for size-dependent morphological categories are highlighted by green areas. The relative distribution of the morphological categories for nascent SSA was adapted from Kaluarachchi et al., 2022.^1^ Copyright 2022 American Chemical Society.

Figure 3A,B shows the size-dependent relative distribution of morphological categories of nSSA and aged SSA within three selected volume-equivalent diameter ranges of 0.10–0.18, 0.18–0.32, and 0.32–0.60 μm, respectively. The statistical probability distribution analysis to assess the significance in the distribution of morphological types across the size ranges was conducted as described in prior studies.^1^ For both sample types, as the particle size decreases, a significant increase in the relative abundance of rounded particles and a concurrent but smaller decrease of core–shells was observed.^1^ Additionally, for each size range, aged SSA had a higher abundance of core–shell particles as compared to nSSA. Moreover, for both samples, a prism-like morphology was predominantly observed at the largest size range. The relative abundances of rod and rod–shell particles were varying with respect to the particle size but without an apparent trend.

**Figure 4:**
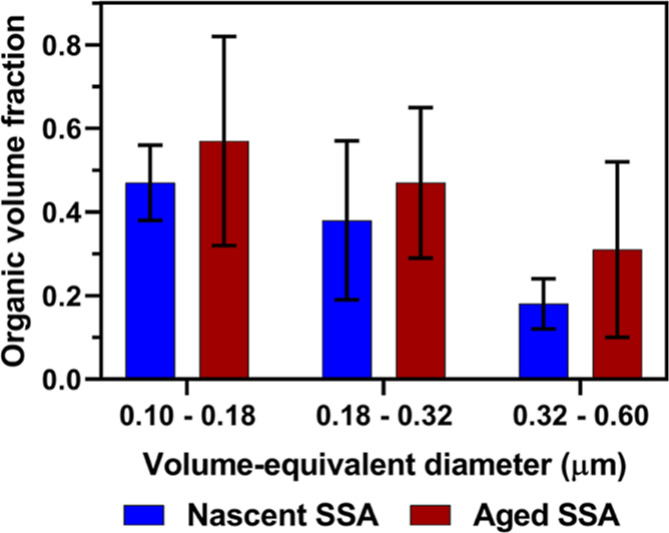
Averaged organic volume fraction measured using AFM at ∼20% RH for core–shell individual SSA from nascent (blue) and aged (brown) samples at three selected volume-equivalent diameter ranges of 0.10–0.18, 0.18–0.32, and 0.32–0.60 μm. Each color bar height and error bar represent the average and one standard deviation, respectively. The OVF for nascent SSA were adapted from Kaluarachchi et al., 2022.^1^ Copyright 2022 American Chemical Society.

Figure 4 and Table 1 show the AFM-based single particle size-dependent organic volume fraction (OVF) and corresponding organic coating thickness (OCT) measurements for core–shell-aged SSA. Additionally, Figure 4 shows the size-dependent OVF values recorded for nSSA core–shells.^1^ Based on the average OVF results, the corresponding average and one standard deviation of OCT were calculated for aged SSA core–shells, and the results were compared with previously recorded data for nSSA.^1^ Overall, as the particle size decreases, the average core–shell OVF for nSSA increased from 0.18 ± 0.06 to 0.47 ± 0.09, while that for aged SSA increased from 0.31 ± 0.21 to 0.57 ± 0.25.^1^ As the OCT values do not display any clear size dependency, the average value over the entire studied size range of 0.1–0.6 μm can be used to assess the effect of aging on the shell thickness. Specifically, the average and one standard deviation of core–shell OCT for nSSA was 16 ± 6 nm, while that for aged SSA was 24 ± 13 nm.

**Table 1:**
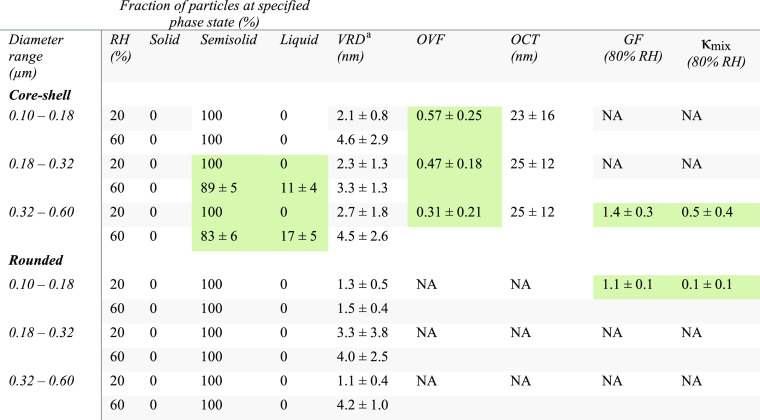
Summary of Core–Shell and Rounded Aged SSA Properties for Three Selected Volume-Equivalent Diameter Ranges of 0.10–0.18, 0.18–0.32, and 0.32–0.60 μm During the Phytoplankton Bloom Including an Averaged and One Standard Deviation for Fraction of Particles at Solid, Semisolid, and Liquid Phase States at 20 and 60% RH, VRD for the Semisolid Shell of Core–shell and Semisolid Rounded Particles, OVF, and Corresponding OCT for Core–shell Particles, Volume-Equivalent GF, and Hygroscopicity Parameter (κ_Mix_)

a Data range reported by probing shell region of core–shell, and the center of rounded aged SSA at the semisolid phase state. Statistically significant differences for a particular property are highlighted by green areas.

As will be demonstrated in the next section using AFM–PTIR spectroscopy, the core and shell regions of core–shell-aged SSA and nSSA are predominantly enriched with inorganic and organic compounds, respectively.^1^ Therefore, the larger OVF and OCT values are indicative of a relatively higher organic content in the core–shell particles.^1,2,18,61^ For both nSSA and aged SSA core–shells, a significant increase of the average OVF with the decreasing particle size was observed, which indicates significant organic enrichment in smaller particles.^1^ In addition, compared to nSSA, aged SSA core–shells showed a significantly higher OVF and OCT values for all sizes, which indicates substantial organic enrichment as a result of atmospheric aging. Collectively, the observed bulk organic mass fraction enrichment in the smaller aged SSA (Figure 1 and corresponding discussion above) could be therefore attributed to a combination of an increase in OVF for smaller core–shell particles and increased abundance of smaller predominantly organic rounded particles. Additionally, the observed increase of bulk organic mass fraction of aged SSA relative to nSSA (Figure 1) is likely due to a significant increase in the abundance of core–shell particles with a higher organic content.

### Changes in Single-Particle nSSA Composition due to Atmospheric Aging

**Figure 5:**
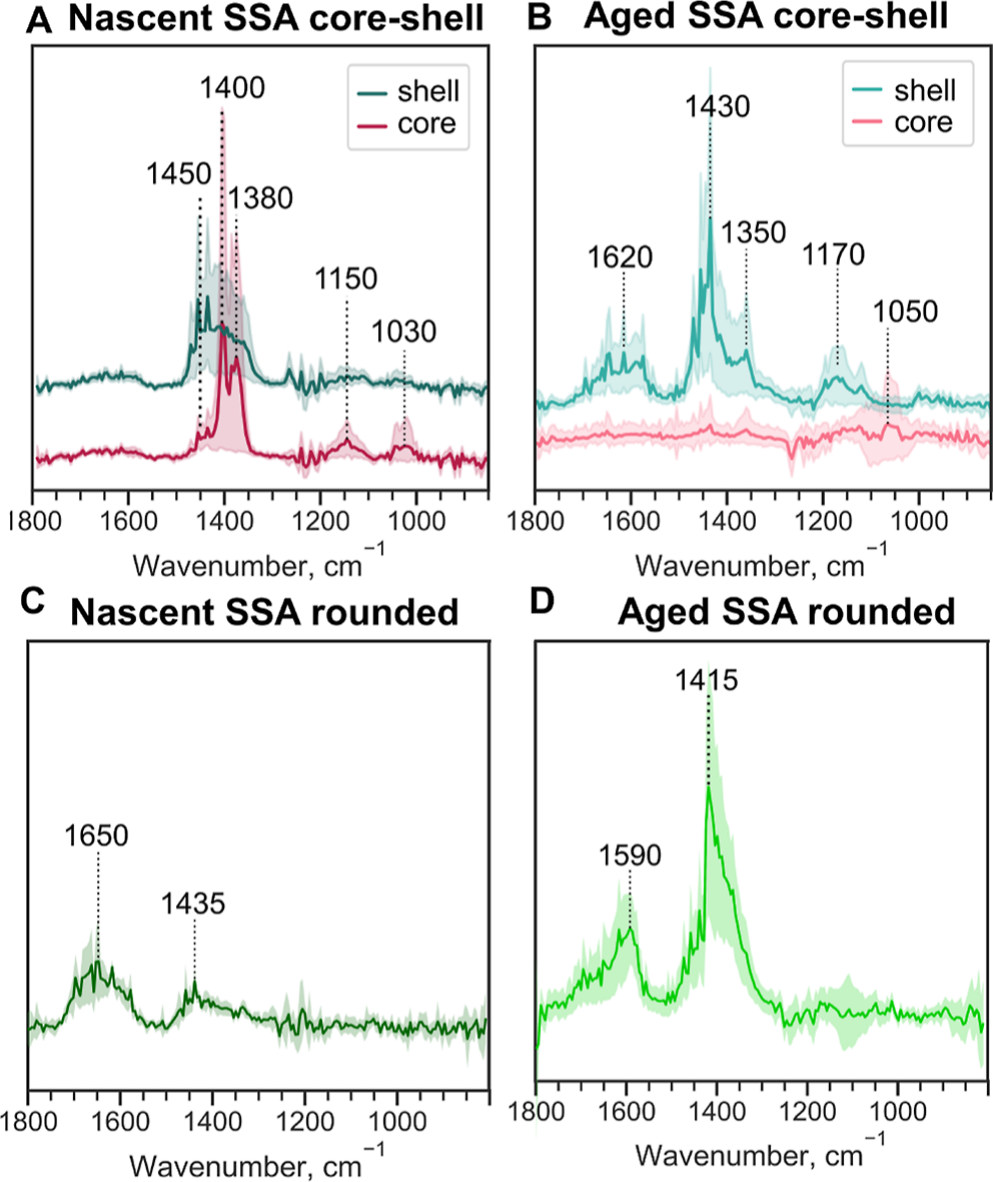
PTIR spectra for (A) nascent and (B) aged core–shell SSA, (C) nascent and (D) aged rounded SSA within the volume-equivalent diameter range of 0.10–0.60 μm. Spectra were taken at core and shell regions for core–shell SSA, and at approximately particle center for rounded SSA. Solid lines show the averaged spectra (number of individual core–shell SSA ≥ 10, and number of individual rounded SSA ≥ 10) and shaded lines represent the 95% confidence interval. The spectra for nascent SSA were adapted from Kaluarachchi et al., 2022.^1^ Copyright 2022 American Chemical Society.

Figure 5A,B shows the AFM–PTIR spectra collected on nSSA and aged SSA core–shell particles at the core and shell regions. The core of nSSA core–shell particles are comprised IR inactive compounds, such as NaCl or contain nitrates [ν_as_(NO_3_^–^): 1400, 1380 cm^–1^], while the shells are enriched with aliphatic-rich compounds [δ(CH_2_, CH_3_): 1450, 1370 cm^–1^].^1^ A small shoulder at δ(CH_2_): 1450 cm^–1^ and modes around ν(C–O, C–C): 1150 and 1050 cm^–1^ are evident in spectra from the nSSA core, suggesting a thin layer of organic coating.^1^ The aged SSA core–shells are spectrally distinct relative to nSSA core–shells. The core of the aged SSA core–shell is largely IR inactive from 800 to 1800 cm^–1^. The shell of aged SSA core–shell is more functionalized in comparison to the nascent shells, as indicated by the larger vibrational mode around 1600 cm^–1^, a broad mode that could have overlapping contributions from ν_as_(COO^–^), ν(C=O), or even amides.^78,79^ Thus, based on the PTIR spectral comparison, the shell region of aged SSA is enriched with more oxygenated organic compounds relative to nSSA and provides a confirmation of the hypothesis that following OH oxidation, oxygenated gases partition to the surface of existing particles whereby the organic fraction increases with aging.

Figure 5C,D shows the PTIR spectra collected on nSSA and aged SSA-rounded particles at the approximate center of individual particles. For both sample types, there are two similar large modes at 1415–1435 cm^–1^ corresponding to aliphatic-rich compounds [δ(CH_2_, CH_3_) and oxygenated functional groups ν(COO^–^)] and around 1600 cm^–1^ that could have overlapping contributions from ν_as_(COO^–^), ν(C=O), or even amides.^78,79^ While relative intensities of these two main modes somewhat differ which may indicate variability of different functional groups, the presence of two modes for both samples suggests similar functional groups are present for both samples. Additionally, the PTIR spectra for rounded aged SSA appear to be spectrally similar to the shell region of core–shell-aged SSA, suggesting the presence of similar functional groups for these samples. We note, due to large chemical diversity within SSA, combined single-particle PTIR spectra show a large variance. Thus, spectral results presented herein demonstrate the presence (or potential absence) of a particular functional group within SSA.^1^

**Figure 6:**
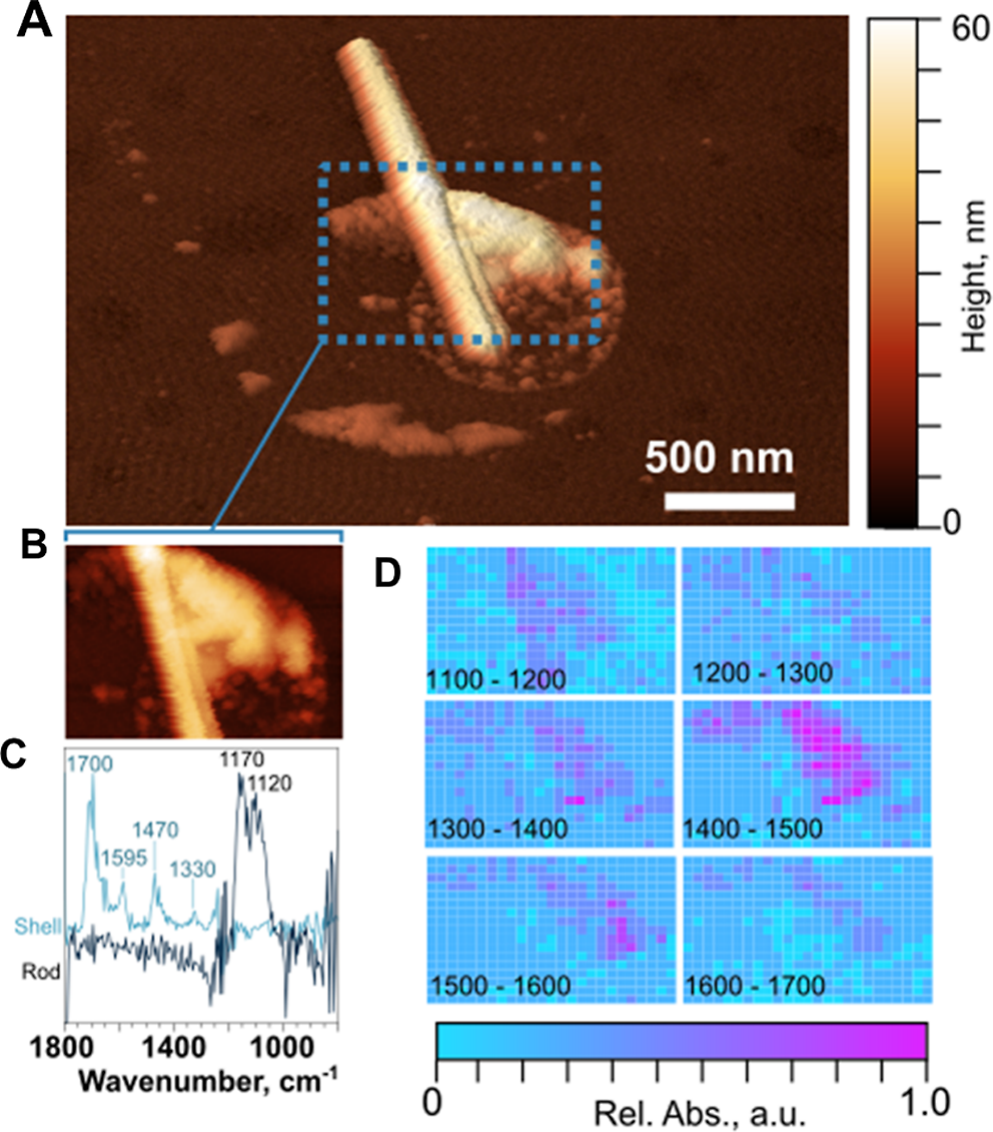
(A) AFM 3D height image and (B) zoomed in region of the aged rod–shell particle with (C) AFM–PTIR spectra measured over the rod (black line) and shell (blue line) regions and (D) hyperspectral maps recorded over the rod–shell particle shown over 1100–1700 wavenumber range with 100 cm^–1^ windows.

Figure 6A,B shows the AFM 3D height image and zoomed in the region for the aged SSA rod–shell particles, where AFM–PTIR spectra were taken. PTIR spectra shown in Figure 6C reveals that the rod is inorganic sulfate, similar to rod particles observed on the nSSA sample,^1^ as evidenced by the ν_as_(SO_4_^2–^) mode at 1170 cm^–1^.^80^ The shell region of rod–shells is organically rich with a distinct mode of ν(C=O) at 1700 cm^–1^.^81,82^ Hyperspectral maps of the particle (Figure 6D) show the spatial distribution of absorbances within 100 cm^–1^ integrated spectral bins. The rod-shaped core of the particle only has absorbances consistent with the sulfate (1100–1200 cm^–1^), while the shell is more intensely absorbing in spectral maps for other spectral regions such as δ(CH_3_, CH_2_): 1330, 1470 cm^–1^, ν_as_(COO^–^): 1570 cm^–1^, ν(C=C): 1595 cm^–1^, and ν(C=O): 1700 cm^–1^. It is likely that the rod–shell particles were formed due to the condensation of semi-volatile or low volatility oxygenated organic compounds on pre-existing rod particles during the heterogeneous aging process in PAM-OFR.^81−83^ Furthermore, the PTIR spectra for the shell region of core–shell- and rod–shell-aged SSA and rounded aged SSA appear to be comparable, suggesting the presence of similar organic functional groups. Formation of more oxygenated organic species in aged SSA core–shells relative to nSSA core–shells could potentially influence particle phase state and hygroscopicity as discussed below.

### Influence of Atmospheric Aging on Phase State and Water Uptake of Rounded and Core–Shell nSSA

Phase state identification on the highest abundance morphologies (i.e., core–shell and rounded) of aged SSA were performed at 20 and 60% RH using the AFM force spectroscopy (i.e., force profiles).^1,11,55,57^ At least five repeated force profiles were collected by probing at the shell region of each core–shell and at an approximate particle center for each rounded particle. The measurements over the core of aged SSA core–shell particles were not reported because it is solid with possibly a thin organic layer, as shown in our prior studies.^1^ The force profiles were then used to quantify VRD (nm, viscoelastic response distance) and RID (ratio of the indentation depth over the particle height) for an individual particle at a particular RH and determine phase states using previously established frameworks based on these measurements.^1,11,55,57^ Previous studies showed that the VRD values can be related to the viscoelastic nature of particles, where higher values generally correspond to lower viscocity.^1,11,57^Table 1 shows the VRD values measured on semisolid particles within three selected volume-equivalent diameter ranges of 0.10–0.18, 0.18–0.32, and 0.32–0.60 μm. A statistical probability distribution analysis on solid, semisolid, and liquid phase states for the shell region of core–shell and rounded aged SSA were performed, as described in previous studies.^1^ Because no apparent size-dependent phase state was observed for core–shells and rounded aged SSA, the phase state results for each particle type were combined over a wider volume-equivalent diameter range of 0.1–0.6 μm.

**Figure 7:**
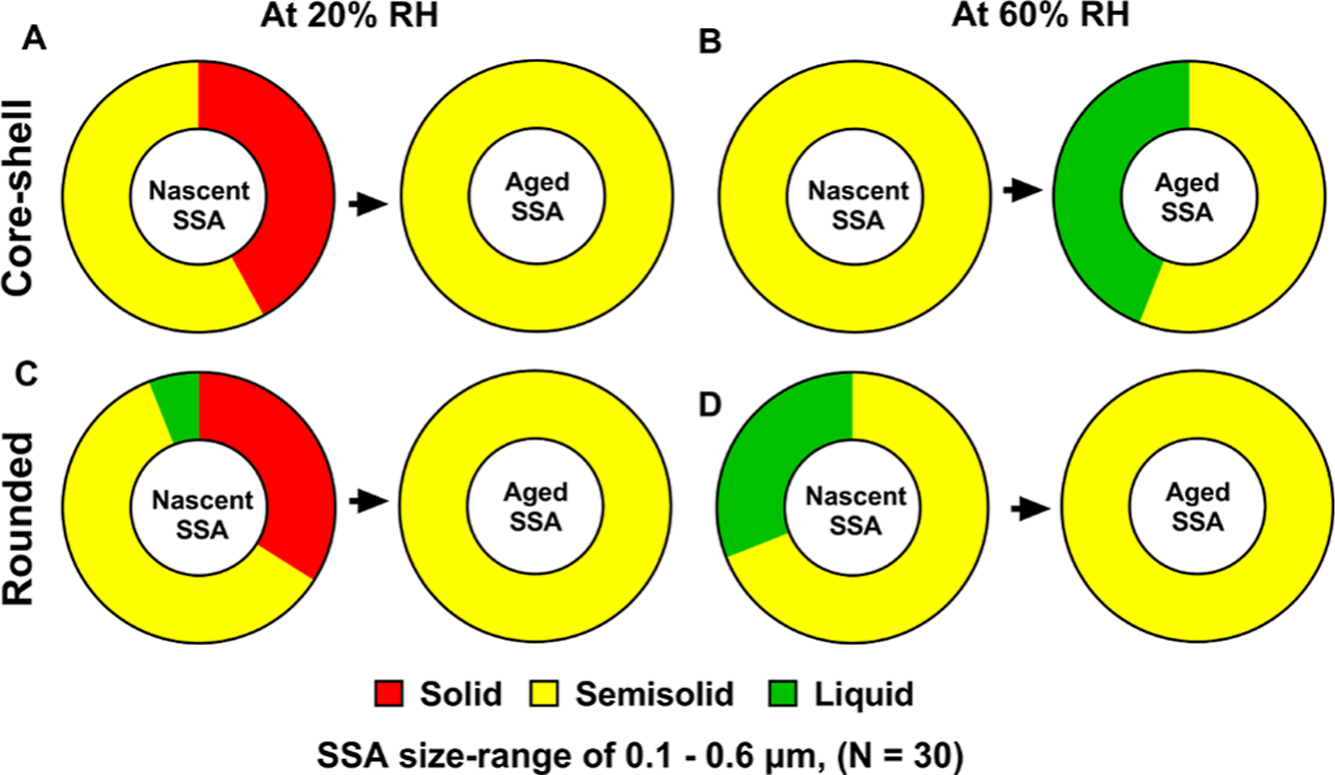
Relative distributions of solid, semisolid, and liquid phase states for (A,B) shell region of nascent and aged SSA core–shell at 20 and 60% RH, respectively (C,D) nascent and aged SSA rounded at 20 and 60% RH, respectively. SSA are within the volume-equivalent diameter range of 0.10–0.60 μm. Arrows are for illustrative purposes only. The phase state information for nascent SSA were adapted from Kaluarachchi et al., 2022.^1^ Copyright 2022 American Chemical Society.

Figure 7A,B shows the relative distributions of solid, semisolid, and liquid phase states for the shell region of aged SSA versus nSSA core–shells. At 20% RH, nSSA had either solid or semisolid shells, while aged SSA had only semisolid shells. Furthermore, the VRD values measured on aged SSA semisolid shells were greater than that for nSSA, which is likely indicative of lower shell viscosity as a result of atmospheric aging.^1^ The results were consistent with the presence of more oxygenated organic compounds, as evident by the PTIR measurements discussed above. As RH increased to 60%, aged SSA shells became hydrated and a significant fraction of shells were liquid, while nSSA shells were only semisolid.^1^ Collectively, due to the aging, the phase state of shells shifted toward a more semisolid state (likely with lower viscosity) within the considered RH range.

Figure 7C,D shows the relative distributions of solid, semisolid, and liquid phase states for rounded aged SSA versus nSSA. Specifically, at 20% RH, rounded nSSAs were either solid or semisolid, while rounded aged SSA were only semisolid.^1^ However, the VRD values measured on semisolid-rounded nSSA versus aged SSA did not show a significant variability, which was consistent with the presence of similar functional groups for each sample, as evident by the PTIR measurements discussed above. As RH increased to 60%, rounded aged SSA only showed a semisolid phase state, while the majority of nSSA rounded was semisolid with a small fraction as a liquid.^1^

The 3D growth factor (GF) and corresponding hygroscopicity parameter (κ_mix_) of core–shell and rounded aged SSA were determined at 80% RH using a previously reported approach, and the corresponding average and one standard deviation values are reported in Table 1.^1,11,67,68,84^ The measurements were performed on the core–shell and rounded aged SSA at the highest relative occurrence size ranges of 0.32–0.60 and 0.10–0.18 μm, respectively. Specifically, the GF (range 1.2–1.7) and κ_mix_ (average 0.5 ± 0.4) values for aged SSA core–shells were higher compared to the GF (range 1.2–1.4) and κ_mix_ (average 0.3 ± 0.2) values of nSSA core–shells for similar sizes as reported by us previously.^1^ An increase in hygroscopicity and water uptake observed on the aged SSA core–shells relative to nSSA core–shells is consistent with the AFM–PTIR spectral data and AFM phase state measurements, which showed formation of more oxygenated organics and increasing the relative abundance of liquid shells as a result of aging. In contrast, there is no apparent difference in the nSSA-rounded particle GF (range 1.0–1.2) and κ_mix_ (average 0.1 ± 0.1) values and aged SSA-rounded particle GF (range 1.0–1.2) and κ_mix_ (average 0.1 ± 0.1) values. These results are consistent with AFM–PTIR measurements described above, where spectra for these two samples suggest the presence of similar functional groups.

## Summary and Implications

Atmospheric aging can alter various physicochemical properties of the SSA.^36−39,74,85,86^ The current study investigated the effects of atmospheric aging of nSSA (i.e., oxidation with OH radicals corresponds to 4–5 days of atmospheric aging) on their size-dependent morphology, composition, water uptake, phase state, and particle-to-particle variability of these properties, for submicron nSSA and aged SSA collected during a mesocosm study. As is evident by filter-based measurements, both nSSA and aged SSA showed an increase in the organic mass fraction with decreasing particle size. In addition, aging further increased the organic mass fraction in aged SSA. These results can be rationalized with complementary single-particle measurements presented here, which showed a relative increase in the abundance of aged SSA core–shells with significantly higher organic coating thickness, compared to nSSA. Additionally, as is evident by PTIR spectra, aged SSA core–shells contained relatively more oxygenated organic species than nSSA. We also noticed a significant particle-to-particle variability in the aged SSA organic content and composition. Aged SSA morphology, organic content, and composition can influence their direct and indirect aerosol effects (e.g., scattering, water uptake, CCN, and IN efficiency).^13,26,87,88^ For example, prior studies showed core–shells can undergo atmospheric aging within days, while rounded particles can take up to weeks or months.^36,52^ The organic coating thickness of core–shells can further control the diffusion time scale of gas phase molecules in the atmosphere, that is, thicker coating can significantly increase the diffusion time into the particle.^36,89^ Moreover, as demonstrated in the current study, aged SSA morphology and organic content can modify their hygroscopicity by the presence of water-soluble or insoluble compounds.^1,11,46,55,62^ In particular, higher hygroscopicity and more efficient water uptake properties were observed for aged SSA core–shells, which had more oxygenated organic species relative to nSSA core–shells, while rounded aged SSA and rounded nSSA had similar water uptake properties and no apparent changes in the composition. The aged SSA morphology and composition-induced water uptake can modify their sizes and affect the direct and indirect aerosol properties.^1−3,11,26,27,68,90^

Atmospheric aging increased the abundance of core–shells at the semisolid or liquid phase state, while nSSA core–shells were primarily solid or semisolid (RH range of 20–60%). The results can be compared with prior reports conducted on aged SSA model systems. For example, a study conducted on model organic aerosols showed a significant enhancement of the particle hygroscopicity upon exposure to OH radicals that were initially hydrophobic.^43,46,49^ Another study showed that the atmospheric aging of model aerosol particles can potentially increase the particle hygroscopicity, thus the formation of more semisolid or liquid particles even at dry RH conditions, which agreed with our observations from the current study.^74,91^ Particle phase state and hygroscopicity can control their indirect aerosol effects, where liquid droplets can be better CCN while solid particles can be better IN.^1,3,11,26,27,55,58,68,92,93^ Thus, aged SSA in the liquid phase state can likely show an enhanced CCN ability compared to nSSA at the solid phase state.^36−39,72^ Furthermore, the particle phase state can alter the bulk diffusion of small molecules (e.g., water, nonvolatile organic species), and characteristic time for their mass-transport and mixing by molecular diffusion in the aged SSA.^74,85,86,89^ For example, diffusion time required for small molecules in a solid particle is much higher (∼years) than that of a semisolid (∼seconds).^76,89,94−96^ As demonstrated in the current study, the VRD values measured on shells of aged SSA core–shells were shifted toward relatively higher values, which likely indicated that the aged SSA shells were becoming less viscous due to the aging.

Overall, our results illustrate that atmospheric aging results in significant changes in SSA morphology, composition, phase state, and water uptake properties. Significantly, these changes are not the same for the entire SSA population but rather show a significant particle-to-particle variability and size-dependency. These findings highlight the importance of single-particle methods that are complementary to bulk ensemble-average approaches and support the premise that future studies aiming to better understand and model the effects of atmospheric aging of SSA should account for possible aerosol size effects and particle-to-particle variability.
